# Biochar and nano-silicon partnership alleviates vanadium toxicity in rice through improving antioxidant defense, nitrogen assimilation and iron plaque formation

**DOI:** 10.3389/fpls.2026.1778126

**Published:** 2026-03-06

**Authors:** Xiaolei Wang, Chuchu Chen, Xiaoxuan Sun, Chuanzhi Wang, Haiying Tang

**Affiliations:** 1 School of Food and Biological Engineering, Suzhou University, Suzhou, Anhui, China; 2 School of Agriculture and Biotechnology, Hunan University of Humanities, Science and Technology, Loudi, China

**Keywords:** biomass, chlorophyll, enzymes activity, nitrogen metabolism, rice, vanadium

## Abstract

**Introduction:**

Biochar (BC) and nano-particles have emerged as promising strategies to mitigate heavy metal toxicity and remediate polluted soils. Vanadium (V) is a toxic metal posing hazardous impacts to plants and humans. The role of BC and nano-particles, particularly their combination to alleviate V toxicity, is poorly understood. Thus, this study explored the role of BC and silicon nano-particles (Si-NPs) partnership in mitigating the V toxicity in rice.

**Methods:**

The study has five treatments: control, V stress (30 mg kg^-1^ soil), V stress (30 mg kg^-1^ soil) + biochar (3%), V stress (30 mg kg^-1^ soil) + Si-NPs (150 mg kg^-1^ soil), and V stress (30 mg kg^-1^ soil) + biochar (3%) + SiO-NPs (150 mg kg^-1^ soil).

**Results:**

The study results revealed that V toxicity decreased rice growth by declining root growth, chlorophyll pigments (78.72-111.50%), nitrogen assimilation, and increasing oxidative stress, membrane damage, and V accumulation in rice plants. Biochar + Si-NPs enhanced rice biomass (20.33%) and grain yield (67.64%) by increasing antioxidant activities (54.12-99.38%), nutrient uptake (58.80-81%), osmolytes synthesis, and decreasing V accretion in rice roots (64.05%) and shoots (91.65%). This increase in rice growth was also linked with an increase in activity of nitrogen assimilation enzymes (nitrate reductase: NR, 65%, glutamine synthetase: GS, 71.82%, glutamate synthase: GOGAT, 106% and glutamate dehydrogenase: GH, 25%) and iron plaque formation.

**Conclusion:**

These findings suggest that the partnership between BC and Si-NPs enhanced root growth, chlorophyll synthesis, antioxidant activity, nitrogen assimilation, and iron plaque formation, while decreasing oxidative damage and V accumulation, thereby increasing plant growth. Thus, a combination of BC and Si-NPs can be an important strategy to mitigate the V toxicity and enhance rice production in V-polluted soils.

## Introduction

Vanadium (V) is a hazardous metal posing toxic impacts on plants, humans, and the ecosystem (Parnell, 2022). It occurs naturally on Earth; nevertheless, mining, fossil fuel burning, and steel production have increased its occurrence in the environment and soils (Zhang et al., 2023). Vanadium has lower mobility and exists in different forms such as V^2+^, V^3+^, V^4+^, and V^5+^; however, V^5+^ is considered to be toxic and a potent oxidizing agent (Rinklebe, 2022). The excessive concentration of V inhibits growth and poses morphological damage to plants (Al-Huqail et al., 2024). The adverse impacts of V are linked to excessive reactive oxygen species (ROS) production, disrupted enzyme activity, and gene expression (Hanus-Fajerska et al., 2021). It also disturbs the cellular process, water and minerals uptake, and photosynthetic efficiency, ultimately decreasing the plant’s growth (Nawaz et al., 2018; Manzoor et al., 2022). The presence of V in the growing medium also changes the plant water status, photosynthetic assimilation, root growth and decreases stomatal length and pore width (Kumar et al., 2022). Moreover, V inhibits carbohydrate metabolism and damages the stomata aperture and guard cells, thus causing growth losses (Basit et al., 2023). Vanadium toxicity also negatively affects soil microbial communities and disrupts soil processes, such as enzyme activity, nitrogen mineralization, nitrification, and microbial community composition (Yang et al., 2014; Wang et al., 2024b). Additionally, higher concentration of V inhibits microbial diversity and disturbs the DNA recombination, which cause apoptosis and reduces microbial abundance (Tang et al., 2022). Therefore, it is crucial to mitigate deleterious impacts of V on plants, for safeguarding human health.

Rice is an important staple crop (Mohidem et al., 2022) with India and China being major rice-producing nations, and China has a share of >50% in the world’s rice production (Li et al., 2023). China is a big producer and user of V minerals, which increases the chances of V entry into agricultural soils (Yang et al., 2017). Rice quickly uptake V, which in turn negatively affects its growth (Mehmood et al., 2021). Vanadium toxicity (35 mg L^–1^) significantly caused reduction in rice growth by inducing oxidative damage, protein synthesis and reducing the photosynthetic efficiency (Altaf et al., 2020). Therefore, considering these issues, it is essential to reduce the V uptake and accretion in rice, guaranteeing safer rice production to meet human needs and safeguard human health.

Biochar (BC) has the ability to counteract heavy metal toxicity, owing to its functional groups and large surface area (Hassan et al., 2025). Biochar absorbs contaminants and reduces their availability in soil and subsequent toxic impacts on plants (Singh et al., 2021; Hassan et al., 2025). Biochar resist microbial decomposition and persists for decade in soil, which makes it an excellent amendment to remediate polluted soils (Kuzyakov et al., 2014). Biochar immobilizes V in soil via different mechanisms, including ion exchange, complexation, and electrostatic interactions (Kończyk et al., 2022). The effectiveness of BC in immobilizing the V-contaminated soils depends on BC properties and chemical forms of V (El-Naggar et al., 2021). Biochar also reduces the V accumulation in plant parts, hydrogen peroxide (H_2_O_2_) and malondialdehyde (MDA) production, and increases antioxidant activity, methylglyoxal activity, and expression of metal-tolerant genes contributing to an increase in plant growth (Altaf et al., 2025). In alfalfa plants, ferrous sulfate modified BC significantly decreased the availability of V by increasing the soil iron and organic matter availability (Aihemaiti et al., 2022).

Nanoparticles (NPs) also gained significant popularity to improve the stress resilience and crop yield (Sun et al., 2022). Nanoparticles have an appreciable ability to mitigate heavy metal toxicity. They bind the toxic metals via hydrogen bonding, electrostatic forces, and coordinating bonding (Xu et al., 2019). Nano-particles (titanium dioxide) have been reported to immobilize V in soils, contributing to an increase in crop growth and yield (Haak et al., 2024). Another study witnessed that SiO-NPs (150 mg kg^-1^) substantially enhanced the photosynthetic efficiency, antioxidant activities, and gene expression in response to V (Altaf et al., 2024). Nano-particles also upsurge phyto-hormones production and change the expression of genes involved in hormones, phenolics, and lignin synthesis, thus increasing the V tolerance (Wang et al., 2024a). These findings indicate BC and NPs can be important strategies to counteract V toxicity. Nevertheless, the role of combined BC and NPs application in mitigating the V is not investigated yet. Therefore, considering these facts, we hypothesized that BC + Si-NPs would more effectively mitigate the V toxicity by modulating plant functioning, nitrogen metabolism, iron plaque formation, and reducing V availability. The aims of study was: to understand the effects of V on growth, plant functioning, and N assimilation ii) to understand impacts of BC and Si-NPs on plant functioning, N assimilation, and crop yield under V stress iii) to determine the impacts of BC and Si-NPs on V partitioning in plant tissues, nutrient acquisition, soil properties and iron plaque formation under V stress.

## Materials and methods

### Experimental site and biochar preparation

The present study was executed at Suzhou University, in open air growth chamber equipped with a rain-protected shelter. The soil was taken from experiment field and sieved to remove debris; thereafter, the pots were filled.

The soil was acidic (5.52 pH) with silt loam texture and carbon contents of 11.20 g kg^-1^, and available phosphorus (AP), available potassium (AK), and total nitrogen (TN) contents of 26.32 mg kg^-1^, 119.29 mg kg^-1^, and 1.88 g kg^-1^, respectively. The plastic pots (32.5 cm × 25.5 cm) were filled with soil (10 kg dry weight), and thereafter, vanadium (30 mg kg^-1^) was thoroughly mixed with the soil and 70% field capacity was maintained and placed in the dark for 60 days. Then, five rice seedlings were transplanted per pot, and an irrigation level of 2–3 cm was kept during the growing period. Rice straw was subjected to pyrolysis at 500 °C under limited oxygen supply with a heating rate of 5 °C _min−1_, and thereafter, it was sieved to measure different properties. Silicon NPs produced by the chemical method (precipitation) were purchased from Shanghai Xiaohuang Nano-technology Co., Limited, China. Biochar and Si-NPs were subjected to scanning electron microscope (SEM), Fourier Transform Infrared Spectroscopy (FTIR: transmittance %), and Energy Dispersive Spectroscopy (EDS). Biochar was alkaline in nature with 9.77, and further details about BC and Si-NPs properties are given in the results section.

### Experiment details

The study was performed in a completely randomized design with three replications. The study contained different treatments: control, V stress (30 mg kg^-1^ soil), V stress (30 mg kg^-1^ soil) + biochar (3%), V stress (30 mg kg^-1^ soil) + Si-NPs (150 mg kg^-1^ soil), and V stress (30 mg kg^-1^ soil) + biochar (3%) + SiO-NPs (150 mg kg^-1^ soil). Biochar was used at a rate of 3% because it showed effective mitigation of V toxicity in earlier studies (Li et al., 2023). A recent study also documented that application of 3% BC effectively mitigates the V toxicity in rice (Altaf et al., 2025). The concentration of nano-particles was selected from earlier findings showing that 150 mg kg^-1^ nano-particles application significantly mitigated the V toxicity (Altaf et al., 2024; Wang et al., 2024a). The Si-NPs were incorporated into soil by mixing in water as suggested by Altaf et al. (2024). The plant samples were taken 40 days after transplanting to measure different physiological and biochemical traits.

### Measurement of photosynthetic pigments, and leaf water contents

For measuring leaf photosynthetic pigments, 0.5 g of fresh leaves were homogenized in a 90% acetone solution. Later mixture was homogenized, and absorbance was measured at 665, 649, and 470 to determine the chlorophyll (Chl) a, Chl-b, and carotenoid (Cart) contents (Arnon, 1949). Leaves were weighed (FW), soaked in water for 24 h, and afterwards removed from water and weighed again (TW). The same leave samples were oven dried (DW), and relative water contents (RWC) were measured as: (FW−DW)/(TW−DW)×100 (Chattha et al., 2022). In the case of anthocyanin, fresh rice leaves (0.5 g) were ground (potassium phosphate buffer: PPB, 5 mL), centrifuged, and the absorbance was read at 600 nm for measuring anthocyanin contents.

### Measurement of oxidative markers

For electrolyte leakage (EL), leaves were collected and placed in water for half an hour having 25 °C temperature, and the first electrical conductivity (EC_1_) was determined. Thereafter, leaves were again incubated in a water-bath for 24 hours, and EC_2_ was measured, and EL was determined as: EC_1_/EC_2_ × 100. For measuring leaf malondialdehyde (MDA) contents, 0.5 g fresh rice leaves were collected and ground in 5 mL of trichloroacetic acid (TCA: 0.1%) solution. Then this mixture was centrifuged (12,000 rpm) for 20 minutes. After this, 5 mL thiobarbituric acid (TBA) was mixed and heated (95 °C) for 30 minutes. Later, absorbance was recorded at 532 and 600 nm for measuring MDA concentration (Rao and Sresty, 2000). For hydrogen peroxide (H_2_O_2_), samples were homogenized and then mixed with 1 M potassium iodide, potassium phosphate, and PPB (100 µL) and incubated for 30 minutes. Then, the absorbance was measured at 390 nm to measure the concentration of H_2_O_2_ (Velikova et al., 2000).

### Measurement of antioxidant and osmo-protectants

The rice fresh leaves were collected and ground in 5 mL of PPB solution. After grinding, the solution was subjected to centrifugation (8,000), and the supernatant was taken, which was used for measuring antioxidant activity. For measuring the activity of ascorbate peroxidase (APX); 100 μL enzyme extract was collected and added with H_2_O_2_, and later absorbance was read at 290 nm (Nakano and Asada, 1981). The activity of catalase (CAT) was measured after mixing the enzyme extract with H_2_O_2_ by the methods of Aebi (1984). For measuring the peroxidase (POD), the solution containing PPB (50 mM), H_2_O_2_ (300 mM), and enzyme extract (100 μL) was prepared, and absorbance was read at 470 nm (Chanes and Mahely, 1996). In case of superoxide dismutase (SOD), we prepared a mixture of 400 µL H_2_O_2_, 50 µL riboflavin, 50 µL nitro blue tetrazolium, 25 µL PPB, and the extract, and absorbance was recorded at 560 nm (Zhang, 1992). The leaf phenolic concentration was measured by the methods of Principato et al. (1987). For this, leaves were collected, and a mixture was made by using the Folin-Ciocalteu reagent technique, and absorbance was read at 740 nm (Principato et al., 1987). To measure phenolic acid concentration, leaves were ground, and the extract was collected. Thereafter, 24 µL extract and 2824 µL sodium nitrite were mixed and heated for five minutes. After that, this mixture was added to aluminum chloride (28 µL), and after 10 minutes, the absorbance was measured at 510 nm. For the measurement of proline, 0.5 g of freshly collected rice leaves was ground using 3% sulfosalicylic acid and centrifuged (10,000 rpm) for 10 minutes. Then, acid ninhydrin was added to the mixture and incubated for 120 minutes, and later, the absorbance was read at 520 nm (Bates et al., 1973). For free amino acids (FA) and soluble proteins (SP), fresh leaves of rice were homogenized using the PPB. To measure the FA, a mixture comprising 1 mL of extract along with 1 mL of ninhydrin and pyridine was made, and FA was determined with Hamilton and Vanslyke’s (1943) method. For soluble proteins (SP), the extract was collected and added to 2 mL Bradford reagent and later absorbance was measured at 595 nm as suggested by Bradford (1976).

### Measurement of nitrogen assimilation enzymes

The activity of nitrogen assimilation enzymes in rice leaves was determined by using a kit provided by Abbkine Biotechnology Co., Ltd., Wuhan, China. The instructions and protocols of the manufacturer were carefully adapted to get reliable results, and all the analysis was performed in triplicate. The activities of NR and GS were measured after reading the absorbance at 340 and 540 nm. Moreover, GOGAT and GH activities were measured after reading absorbance at 340 nm.

### Measurement of yield traits, tissue nutrients and soil properties

The plants were carefully uprooted at the maturity stage to measure roots and shoots length and biomass. Five plants from each pot were taken for measuring plant height, panicles/plant, and panicle length. The plants were weighed to note biomass yield (BY) and threshed to measure grain yield (GY). The rice plant samples were dried and digested after adding HNO_3_, H_2_SO_4_, and HClO_4_ in an 8:1:1 ratio. Thereafter, samples were further diluted, and the V concentration was measured with an atomic absorption spectrophotometer. For measuring plant nutrient contents, the samples were digested by using HNO_3_ and HClO_4_ (2:1), and nitrogen (N), phosphorus (P), and potassium (K) in rice seedlings were estimated with a Kjeldahl, spectrophotometer and flame photometer techniques. For measuring soil pH, a solution comprising soil and water in a 1:5 ratio was made, and pH was determined with a pH meter. Moreover, soil TN, AP, and AK were estimated with a Kjeldahl, spectrophotometer and flame photometer techniques. The soil samples were digested by HNO_3_, HClO_4_, and HF following a 1:1:2 ratio, and after digestion, the solution was diluted, and V was measured with an atomic absorption spectrophotometer (AAS). For measuring iron plaque, a mixture of ascorbic citrate acetic (ACA) was made by combining ascorbic acid (3 g), sodium acetate (10%, 5 mL), and sodium citrate (0.3 M, 40 mL). Then, 1 g of root samples was collected and placed in this solution and subjected to shaking for a period of 3 hours at 25 °C. The mixture was filtered into flasks, and the washing of roots were done 3 times, and the eluent was taken and placed in the same flasks. The final volume in each flask increased to 100 mL, and Fe and V contents were determined with an AAS.

### Statistical analysis

The data of different traits were subjected to one-way ANOVA with the help of Statistix 8.1. Tukey’s HSD test (*p ≤ 0.05*) was used for multiple comparisons to determine significant differences between means. Furthermore, figures were made with Sigmaplot-10, while trait relationships were determined by using R-studio. The suitability of the data to perform PCA was confirmed via using Bartlett’s Test. The principal components (PC) were collected from centered and scaled variables, and only PC with eigenvalues > 1 were retained.

## Results

### Characterization of nano-particles and biochar

**Figure 1:**
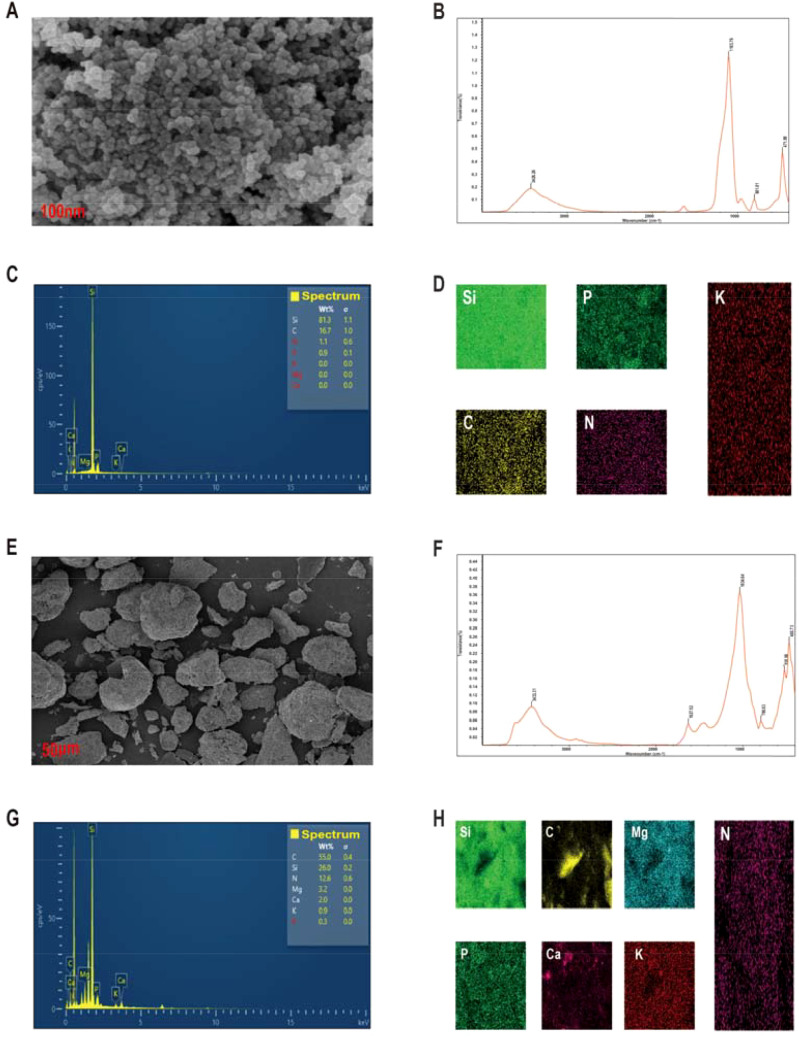
Scanning electron microscopic and energy-dispersive X-ray spectroscopy, and fourier transform infrared analysis of SiO-NPs **(A–D)** and biochar **(E–H)**.

The SEM analysis of Si-NPs showed that NPs are orderly organized with a brief aggregation (**Figure 1**). The EDS analysis showed that Si-NPs contained 81.26% Si, 16.69% C, 1.14% N, 0.87% P, and 0.03% K (**Figure 1**; **Supplementary Table S1**). Additionally, FTIR results showed the peaks at 3426.26 and 1103.76 cm^-1^ (transmission), indicating the presence of oxygen and silicone in the tested specimens (**Figure 1**: Ulhassan et al., 2023). BC made from the rice showed a porous structure (**Figure 1**), indicating its appreciable potential to mitigate heavy metal toxicity (Noor et al., 2025). FTIR analysis of BC showed the different peaks at 3433.31, 1627.52, 1034.64, and 790.63 cm^-1^ (transmission) indicating the presence of O-H, C=O, C=C and C-H groups and these groups play a crucial role in mitigating the metals toxicity (Armynah et al., 2018). The EDS analysis showed that BC contained 54.95% C, 12.61% N, 3.23% Mg, 25.98% Si, 0.26% P, 0.93% K, and 2.03% Ca (**Figure 1**; **Supplementary Table S1**).

### Growth and morphological traits

The study findings showed that V toxicity markedly decreased rice productivity (**Table 1**). Biochar and Si-NPs alleviated the V-induced reduction in growth and yield characters of rice. Notably, under V stress, the combined application of BC + Si-NPs boosted the root length (RL), root fresh weight (RFW), root dry weight (RDW), and plant height (PH) by 39.79%, 34.06%, 44%, and 25.43% respectively (**Table 1**). The impact of all the treatments on tillers/plant was found non-significant (**Table 1**), while V toxicity caused a significant decrease in thousand grain weight (TGW), BY, GY, and harvest index (HI) of rice plants. Biochar and Si-NPs and their integrated supplementation decreased the adversities of V and caused an increase of 31.43%, 20.33%, 67.64%, and 39.13% respectively, in TGW, BY, GY, and HI under V stress (**Table 1**).

**Table 1:** Effects of biochar and silicon nano-particles on growth and yield characters of rice grown in vanadium polluted soil.

Treatments	RL (cm)	RFW (g)	RDW (g)	PH (cm)	TPP	TGW (g)	BY (g/pot)	GY (g/pot)	HI (%)
Control	51.65a ± 1.61	10.66a ± 0.44	4.37a ± 0.29	105a ± 3.27	11	25.56a ± 1.25	197.33a ± 5.56	42.63a ± 1.33	21.64a ± 1.24
V	33.60d ± 1.75	6.19e ± 0.12	2.50e ± 0.09	76d ± 3.28	8	16.89d ± 0.18	155.67d ± 4.64	22.87e ± 1.28	14.72d ± 1.17
V + BC	41.93bc ± 0.38	8.24c ± 0.17	3.16bc ± 0.08	92bc ± 2.16	9	20.21bc ± 0.80	181.00bc ± 4.32	32.20c ± 0.78	17.81bc ± 0.80
V + Si-NPs	38.60cd ± 0.42	7.26d ± 0.21	2.86cd ± 0.06	85cd ± 2.87	9	18.21cd ± 0.17	170.00cd ± 4.08	28.33d ± 0.88	16.68c ± 0.84
V + BC+ Si-NPs	46.97ab2.42	9.31b ± 0.32	3.62b ± 0.03	95ab ± 3.30	11	22.20b ± 0.82	187.33ab ± 4.99	38.34b ± 0.69	20.48ab ± 0.80

The presented data in the table is average of three replicates with ± SD and different letters showing the difference at p < 0.05 according to Tukey test (p < 0.05). RL, root length; RFW, root fresh weight; RDW, root dry weight; PH, plant height; TPP, tillers/pot; TGW, 1000 grain weight; BY, biological yield; GY, grain yield; HI, harvest index; V, vanadium; BC, biochar; Si-NPs, silicon nano-particles.

### Leaf water contents and photosynthetic pigments

Vanadium significantly decreased the leaf RWC and photosynthetic pigments (**Table 2**). The results depicted that compared to control, plants grown in V-polluted soil showed a decrease of 56.37%, 111.50%, 78.72%, 90.43%, and 61.98% respectively, in RWC, Chl-a, Chl-b, Cart, and anthocyanin contents (**Table 2**). Under V stress, plants receiving BC and Si-NPs showed a considerable increase in the aforementioned traits. Nevertheless, BC + Si-NPs application resulted in a marked increase of 44.95%, 72.56%, 42.55%, 64.78%, and 38.59% respectively, in RWC, Chl-a, Chl-b, Cart, and anthocyanin in **Table 2**.

**Table 2:** Effects of biochar and silicon nano-particles on leaf water contents and photosynthetic pigments of rice grown in vanadium polluted soil.

Treatments	RWC (%)	Chl-a (mg g^-1^ FW)	Chl-b (mg g^-1^ FW)	Cart. mg g^-1^ FW)	Anth. (mg g^-1^ FW)
Control	78.00a ± 2.94	2.39a ± 0.13	1.68a ± 0.11	4.38a ± 0.29	8.52a ± 0.30
V	49.67d ± 2.05	1.13d ± 0.07	0.94d ± 0.045	2.30d ± 0.13	5.26d ± 0.18
V + BC	65.33bc ± 2.86	1.60c ± 0.11	1.16bc ± 0.041	3.41bc ± 0.08	6.37c ± 0.19
V + Si-NPs	61.00c ± 0.81	1.49c ± 0.045	1.07c ± 0.035	3.05c ± 0.11	6.05c ± 0.11
V + BC+ Si-NPs	72.00ab ± 2.44	1.95b ± 0.068	1.34 ± 0.086	3.79b ± 0.10	7.29b ± 0.11

The presented data in the table is average of three replicates with ± SD and different letters showing the difference at p < 0.05 according to Tukey test (p < 0.05). Chl, chlorophyll; Cart, carotenoid; Anth, anthocyanin; V; vanadium; BC, biochar; Si-NPs, silicon nano-particles.

### Oxidative markers, osmolytes and antioxidants activity

**Figure 2:**
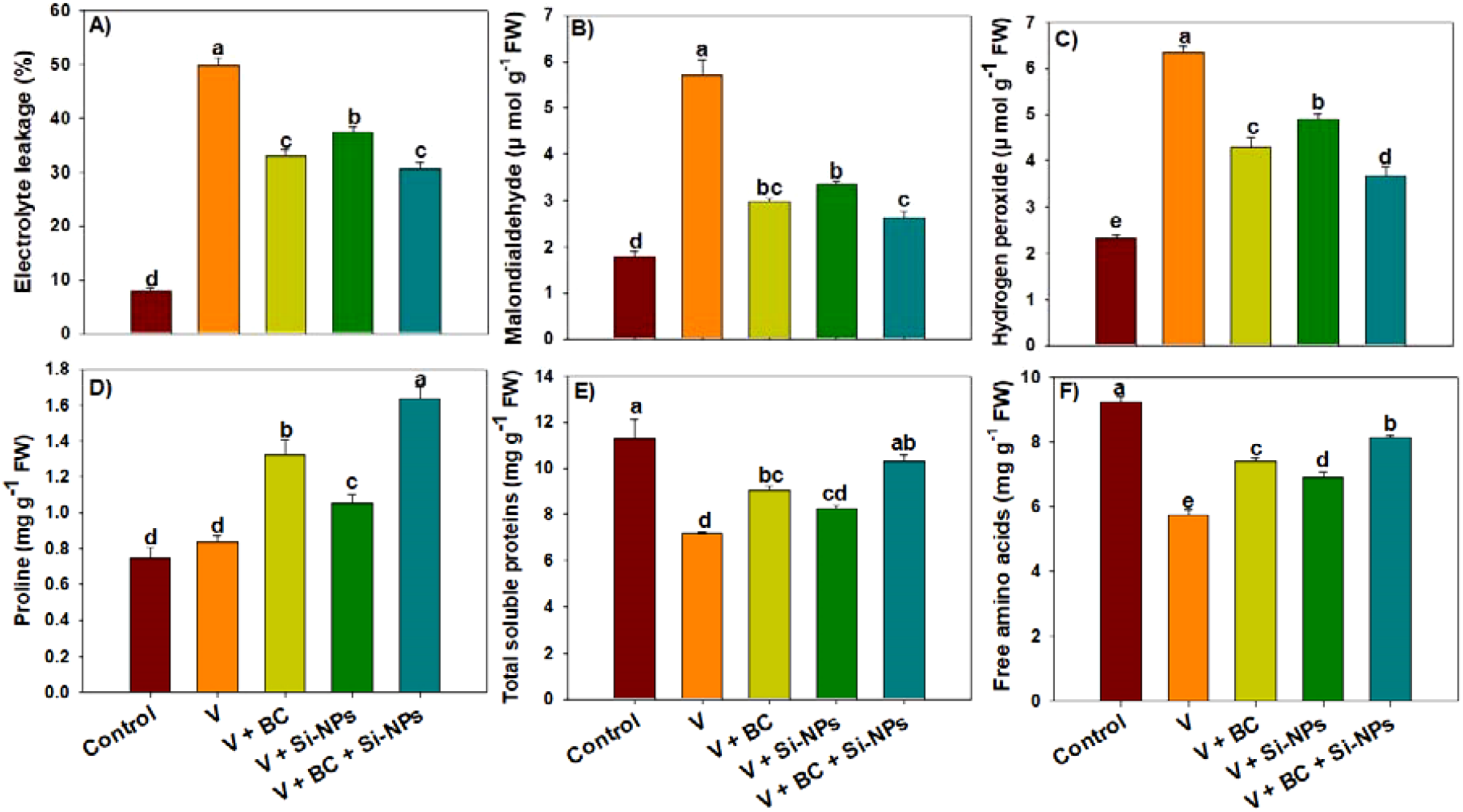
Effects of biochar and silicon nano-particles on stress markers **(A–C)** and osmolytes synthesis **(D–F)** in rice plants grown in vanadium polluted soil. The presented data in the figures is average of three replicates with ± SD and different letters showing the difference at *p < 0.05* according to Tukey test (*p < 0.05*). V, vanadium; BC, biochar; Si-NPs, silicon nano-particles.

**Figure 3:**
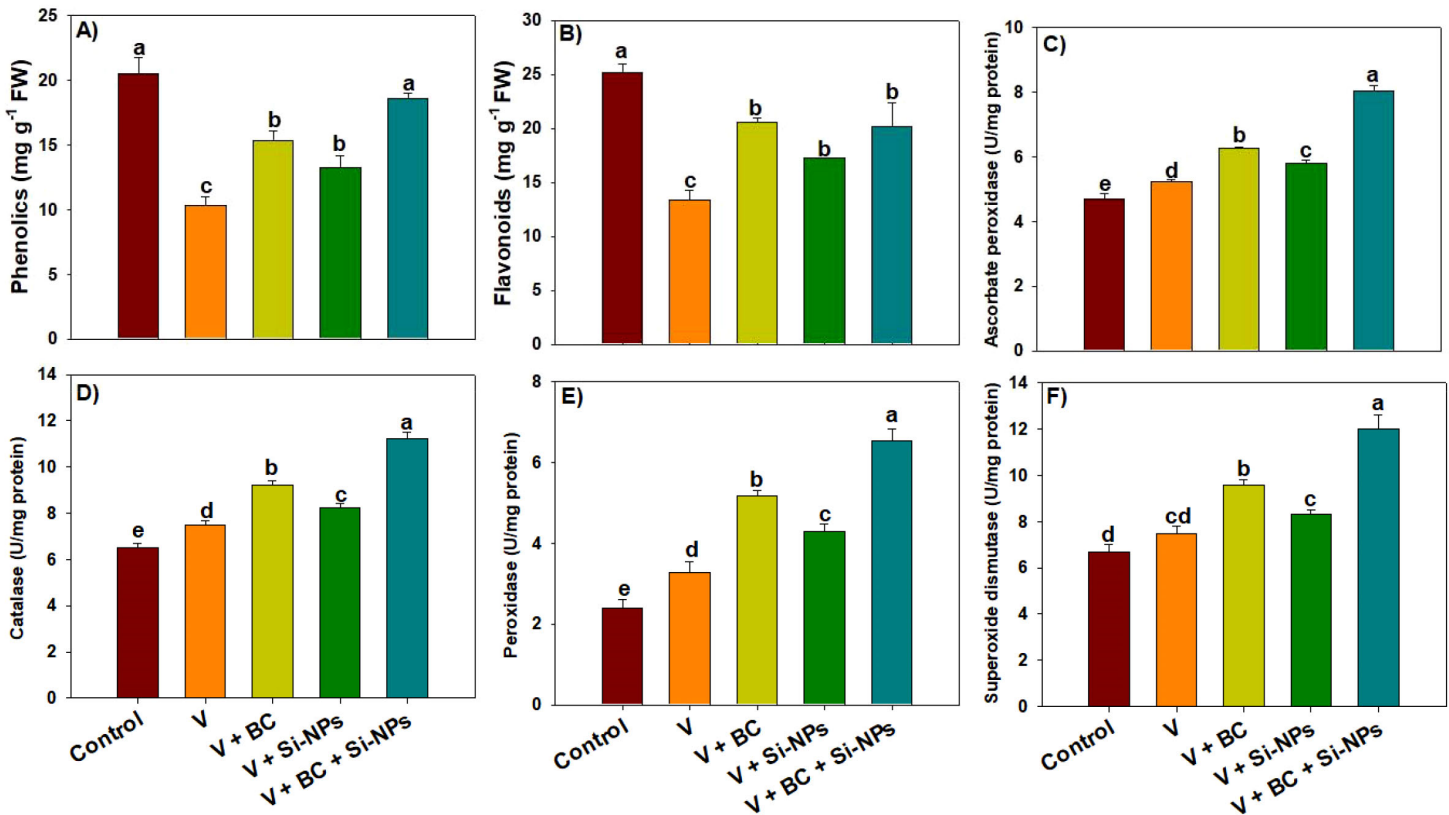
Effects of biochar and silicon nano-particles on phenolics **(A)**, flavonoids **(B)** and antioxidants activities **(C–F)** of rice plants grown in vanadium polluted soil. The presented data in the figures is average of three replicates with ± SD and different letters showing the difference at *p < 0.05* according to Tukey test (*p < 0.05*). V, vanadium; BC, biochar; Si-NPs, silicon nano-particles.

The production of EL, MDA, and H_2_O_2_ increased in response to V stress (**Figure 2**). Biochar and Si-NPs decreased the V-mediated increase in oxidative stress markers (**Figure 2**). We observed that the combination of BC + Si-NPs markedly decreased the EL, MDA, and H_2_O_2_ production by 63.15%, 116.73%, and 72.08% respectively, under V stress (**Table 1**). Proline production was increased under V stress; interestingly, the synthesis of both FA and SP was decreased under V stress (**Figure 2**). Biochar and Si-NPs enhanced proline, FA, and SP under V, which helped in counteracting the V toxicity (**Figure 2**). Notably, plants under V receiving the BC + Si-NPs showed an increase of 94.04%, 43.71%, and 41.21% respectively, in proline, FA, and SP than plants receiving no BC + Si-NPs application (**Figure 2**). The synthesis of both phenolic and flavonoids was also decreased under V stress (**Figure 3**); conversely, BC and Si-NPs significantly enhanced the phenolic and flavonoid synthesis (**Figure 3**). Particularly, the combination of BC + Si-NPs significantly enhanced the phenolic and flavonoid synthesis by 80.17% and 50.74% respectively, under V stress (**Figure 2**). The plant facing the V stress increased the antioxidant activity compared to the control to counter V toxicity. Biochar + Si-NPs applied to V-polluted soil enhanced the activity of APX, CAT, POD, and SOD by 54.12%, 50.60%, 99.38%, and 61.26% in rice plants facing the V stress (**Figure 3**).

### Enzymes activity related to nitrogen metabolism

**Figure 4:**
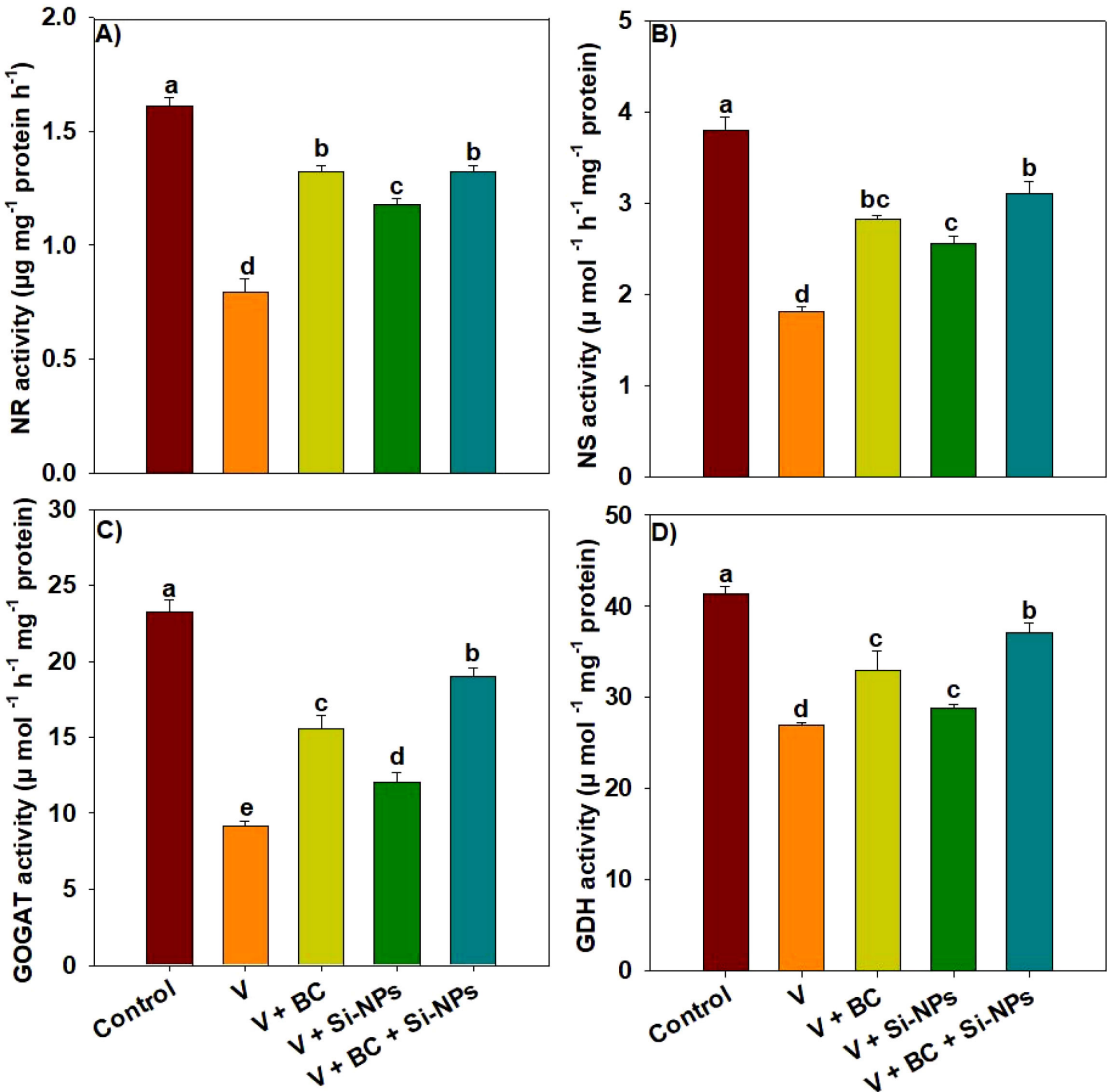
Effects of biochar and silicon nano-particles on activities of nitrogen assimilation enzymes **(A–D)** of rice plants grown in vanadium polluted soil. The presented data in the figures is average of three replicates with ± SD and different letters showing the difference at *p < 0.05* according to Tukey test (*p < 0.05*). V, vanadium; BC, biochar; Si-NPs, silicon nano-particles.

The activity of N metabolism enzymes, including NR, GS, GOGAT, and GDH, responded differentially to different treatments (**Figure 4**). Vanadium toxicity significantly decreased the NR, GA, GOGAT, and GDH activities by 101%, 110%, 153%, and 44.73% compared to the control (**Figure 4**). Treatments with BC + Si-NPs showed a significant increase in NR, GS, GOGAT, and GDH under V stress (**Figure 4**). We observed that BC + Si-NPs significantly enhanced the NR, GS, GOGAT, and GDH activities by 65%, 71.82%, 106%, and 25% under V stress (**Figure 4**).

Biochar and Si-NPs diminished the V accumulation in rice roots and shoots by 64.05% and 91.65% respectively (**Table 3**). Vanadium declined N, P, and K accumulation in rice plants (**Table 1**). Biochar and Si-NPs decreased the competition between nutrients and V and caused a marked increase in nutrient accumulation in rice plants (**Table 3**). We found that the combination of BC + Si-NPs significantly enhanced the N, P, and K concentration by 35.29%, 55.51%, and 55.06% respectively, in rice plants facing the V stress (**Table 3**). Different treatments also significantly affected Si accumulation in rice roots and shoots (**Supplementary Table S2**). Overall, the maximum Si concentration in rice roots (9.24 mg kg^-1^) and shoots (5.11 mg kg^-1^) under V stress was observed with combined BC + Si-NPs application (**Supplementary Table S2**).

**Table 3:** Effects of biochar and silicon nano-particles on vanadium and nutrients accumulation in rice seedlings grown in vanadium polluted soil.

Treatments	Root V (mg kg^-1^)	Shoot V (mg kg^-1^)	Plant N concentration (mg kg^-1^)	Plant P concentration (mg kg^-1^)	Plant K concentration (mg kg^-1^)
Control	-	-	47.33a ± 2.85	34.37a ± 0.78	47.78a ± 1.66
V	15.70a ± 0.54	9.64a ± 0.37	31.34d ± 2.05	21.20c ± 0.82	27.73d ± 1.51
V + BC	11.51c ± 0.49	6.69c ± 0.20	39.07bc ± 1.84	29.20b ± 0.82	39.28b ± 0.80
V + Si-NPs	13.23b ± 0.73	8.45b ± 0.16	32.93cd ± 1.73	27.13b ± 0.75	34.25c ± 1.68
V + BC+ Si-NPs	9.57d ± 0.41	5.03d ± 0.17	42.40ab ± 1.63	32.97a ± 1.70	43.10b ± 0.86

The presented data in the table is average of three replicates with ± SD and different letters showing the difference at p < 0.05 according to Tukey test (p < 0.05). V, vanadium; N, nitrogen; P, phosphorous; K, potassium; V, vanadium; BC, biochar; Si-NPs, silicon nano-particles.

### Iron plaque formation on root surface

**Figure 5:**
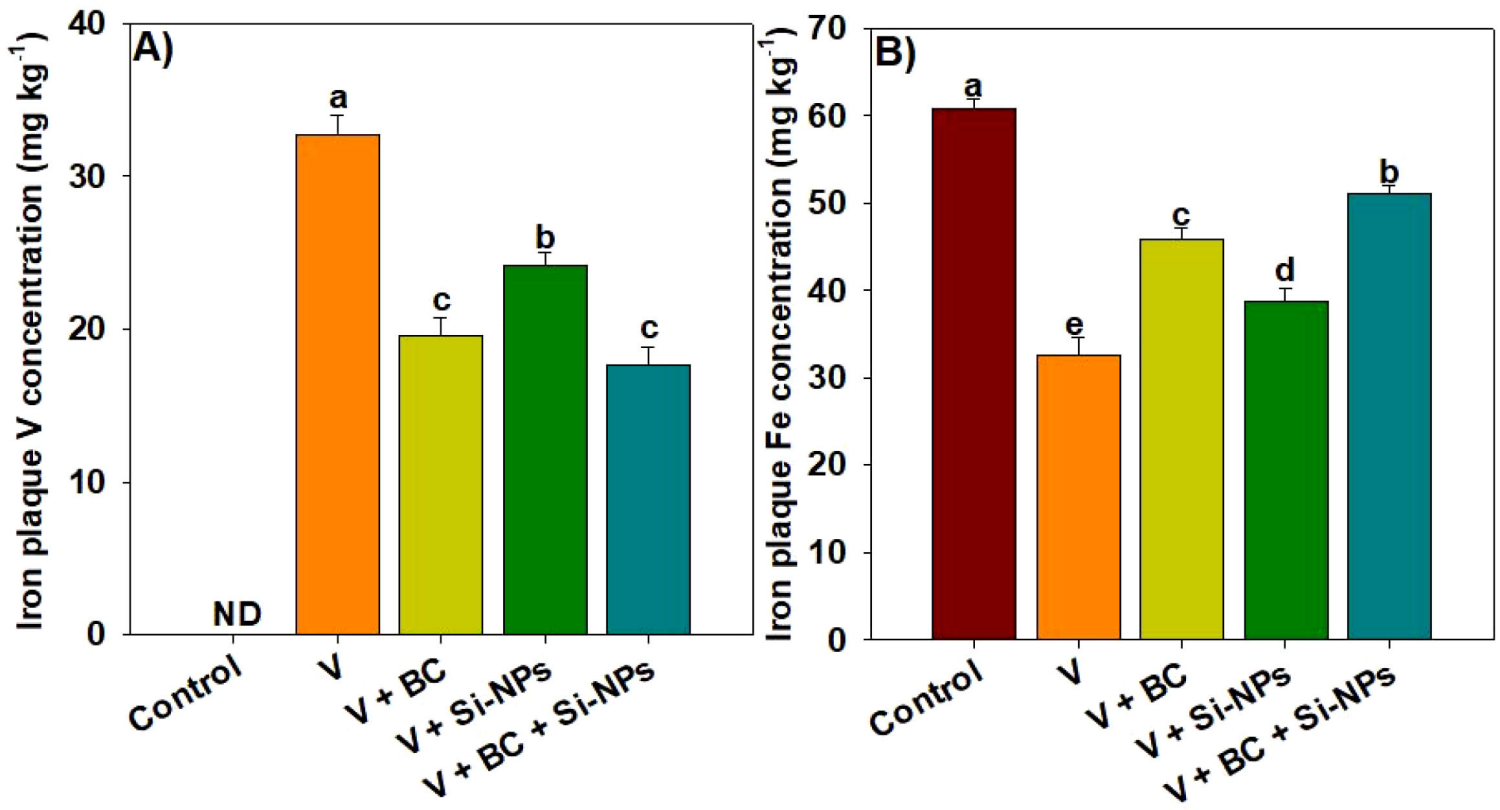
Effects of biochar and silicon nano-particles on vanadium **(A)** and iron concentration **(B)** in ACA extract of rice roots grown in vanadium polluted soil. The presented data in the figures is average of three replicates with ± SD and different letters showing the difference at *p < 0.05* according to Tukey test (*p < 0.05*). V, vanadium; BC, biochar; Si-NPs, silicon nano-particles.

The results of V and Fe in ACA extracts showed that V concentration was markedly increased in rice plant roots planted in V-contaminated soil. The supplementation of BC and Si-NPs significantly decreased the V accumulation in ACA extracts. We observed that BC, Si-NPs, and a combination of BC + Si-NPs showed a reduction of 67.34%, 35.12%, and 85.05% respectively, in V concentration in ACA extract (**Figure 5**). Conversely, BC and Si-NPs enhanced the availability of Fe in the ACA extract, and we found that BC + Si-NPs showed an increase of 56.91%, 47.79%, and 86.14% respectively, in the ACA extract (**Figure 5**).

### Soil vanadium and nutrients availability

The availability of soil V was significantly curbed by application of all the amendments (**Table 4**). Biochar, Si-NPs, and their combination significantly decreased the soil V availability by 62.44%, 43.16%, and 24.69%, respectively (**Table 4**). Silicon, NPs showed minor effect on soil pH, while BC+ Si-NPs showed better increase in soil pH (**Table 4**). Vanadium toxicity significantly decreased the soil nutrient availability. Vanadium stress decreased the soil TN, AP, and AK availability by 81%, 49.16%, and 58.80%, respectively, compared to CK (**Table 4**). Conversely, BC and Si-NPs augmented soil TN, AP, and AK availability 70.90%, 45.07%, and 48.84%, respectively, in V contaminated soil (**Table 4**).

**Table 4:** Effects of biochar and silicon nano-particles on soil vanadium and nutrients availability in vanadmium polluted soil.

Treatments	Soil V (mg kg^-1^)	Soil pH	Soil TN (mg kg^-1^)	Soil AP (mg kg^-1^)	Soil AK (mg kg^-1^)
Control	-	5.58bc ± 0.022	2.00a ± 0.037	33.19a ± 0.81	134.87a ± 2.77
V	23.88a ± 1.25	5.48c ± 0.033	1.10c ± 0.041	22.25d ± 0.68	84.93d ± 3.76
V + BC	16.68bc ± 1.18	5.66b ± 0.033	1.78b ± 0.017	30.29bc ± 0.72	124.15b ± 1.65
V + Si-NPs	19.15ab ± 0.86	5.63b ± 0.060	1.76b ± 0.058	28.31c ± 0.77	112.31c ± 1.79
V + BC+ Si-NPs	14.70c ± 0.43	5.87a ± 0.041	1.88b ± 0.082	32.28ab ± 0.85	126.41b ± 2.07

The presented data in the table is average of three replicates with ± SD and different letters showing the difference at p < 0.05 according to Tukey test (p < 0.05). TN, total nitrogen; AP, available phosphorus; AK, available potassium; V, vanadium; BC, biochar; Si-NPs, silicon nano-particles.

### Trait inter-relationship and structural equation modeling

**Figure 6:**
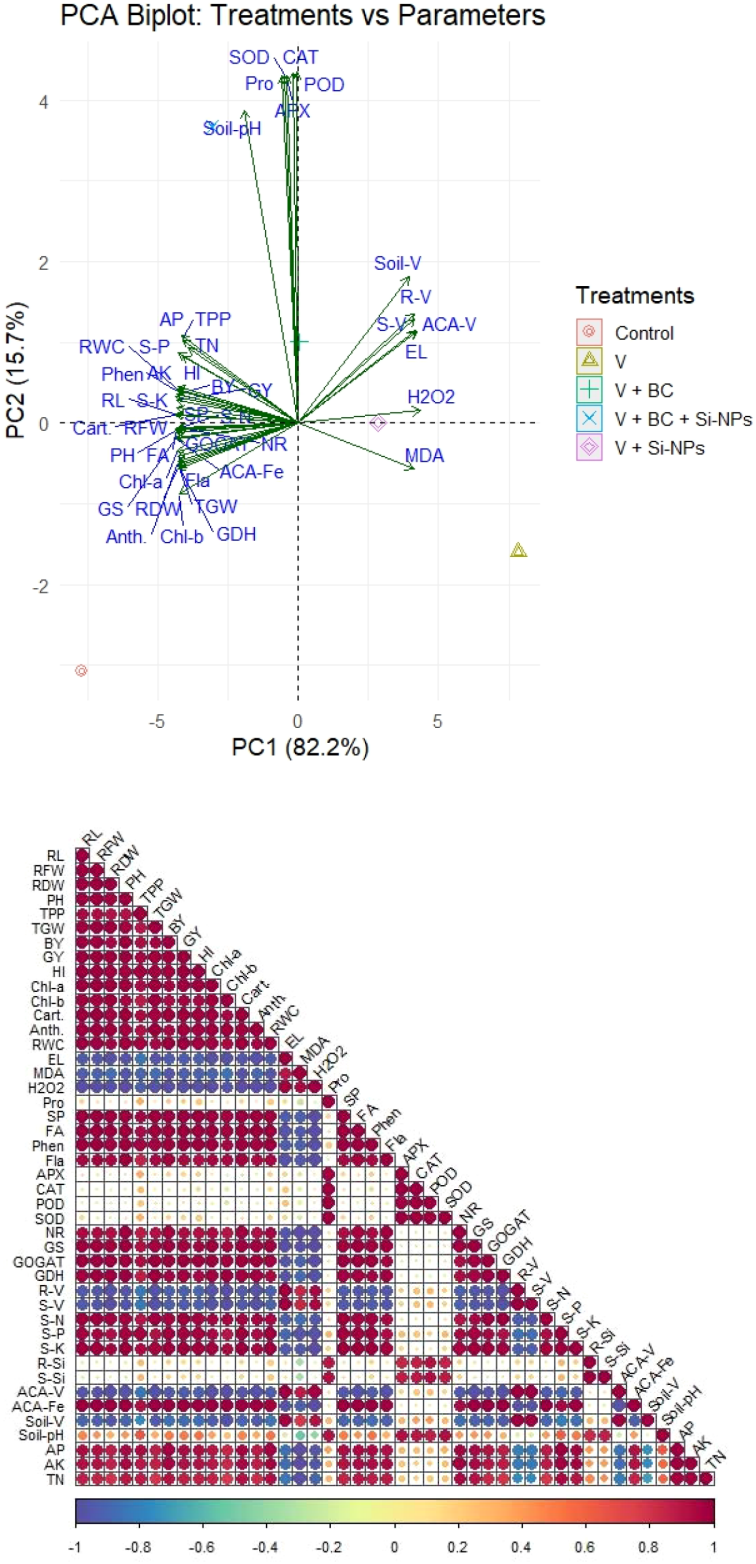
Principal component and correlation analysis for the effects of different treatments on studied parameters. RL, root length; RFW, root fresh weight; RDW, root dry weight; PH, plant height; TPP, tillers/plant; TGW, 1000-grain weight; BY, biological yield; GY, grain yield; HI, harvest index. Chl, chlorophyll; Cart, carotenoids; RWC, relative water contents; EL, electrolyte leakage; MDA, malondialdehyde; H2O2, hydrogen peroxide; Pro, proline; APX, ascorbate peroxide; CAT, catalase; POD, peroxidase; SOD, superoxide dismutase; TN, total nitrogen; AP, available phosphorous; AK, available potassium.

The results of the PCA analysis show that two components have a total variance of 97.9%, with PC_1_ accounting for 82.2% and PC_2_ for 15.7%. The control treatments were located on the negative side of PC_1_, demonstrating the increase in oxidative stress markers. The addition of BC and Si-NPs mitigated the production of oxidative markers by enhancing antioxidant activity. The combined treatments of BC + Si-NPs were clustered on the positive side, showing more positive impacts on plant growth via decreased oxidative stress and enhanced plant functioning and N assimilation (**Figure 6**). The growth traits showed a positive link, indicating their combined effects in increasing the plant performance. The oxidative stress markers showed a negative link with physiological traits, growth traits, and nitrogen assimilation. Moreover, V concentration in roots showed a negative association with nutrients, suggesting a strong competition between V and nutrients at uptake sites. The soil nutrients showed a positive linkage with nutrients, plant functioning and plant growth while negative relationship with V concentration (**Figure 6**).

**Figure 7:**
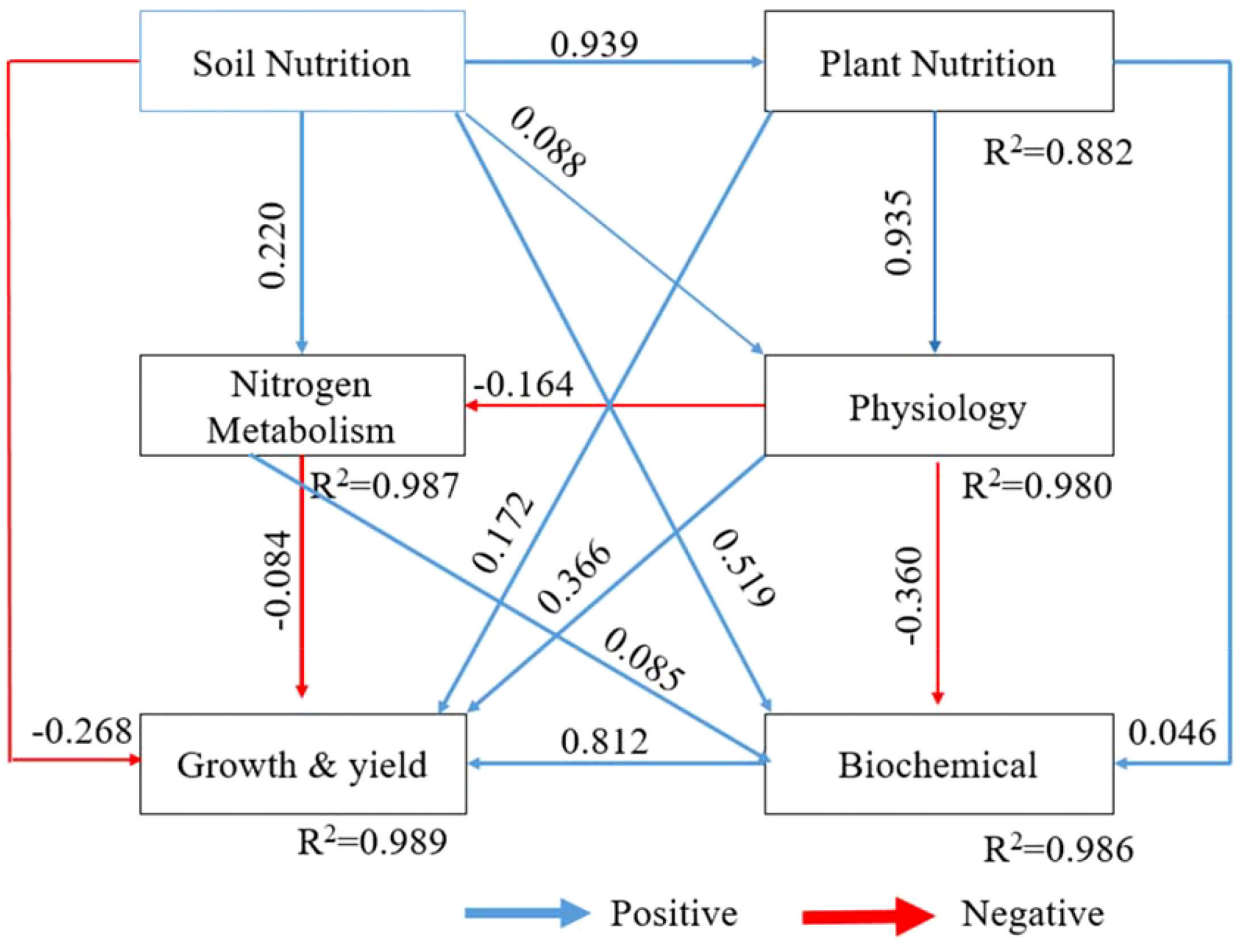
Structural equation model showing the effects of treatments on growth and yield, soil nutrition, physiology, biochemical responses, nitrogen assimilation, and seedling nutrient traits. Values on arrows represent path coefficients (β), and values within constructs indicate R².

To unravel the direct and indirect drivers of crop growth and yield, we used structural equation modeling (SEM) to integrate soil nutrition, plant nutrition, nitrogen metabolism, physiology, and biochemical traits into a single causal framework (**Figure 7**). The model showed that soil and plant nutrition shaped productivity mainly through physiological and biochemical pathways. Soil nutrition had a very strong positive effect on plant nutrition (β = 0.939) and smaller positive effects on nitrogen metabolism, physiology, and biochemical traits, but a direct negative effect on growth and yield (β = −0.268), indicating that its benefits were largely indirect. Plant nutrition strongly improved physiology (β = 0.935) and had a modest positive effect on growth and yield (β = 0.172). Physiology acted as a key intermediary, positively regulating biochemical traits (β = 0.519) but showing a negative direct effect on growth and yield (β = −0.360), suggesting a trade-off between stress responses and final productivity. Biochemical traits were the strongest positive driver of growth and yield (β = 0.812). Overall, the model explained nearly all variation in growth and yield (R² = 0.989), showing that productivity is governed primarily by indirect effects of soil and plant nutrition mediated through physiological and biochemical processes.

## Discussion

Vanadium stress significantly decreased the rice growth and yield, aligning with previous studies (Altaf et al., 2021). This decrease in growth arises from V mediated suppression of root growth and nutrient uptake and is coupled with increased oxidative damages (Chen et al., 2021; Altaf et al., 2021). Notably, V toxicity decreased root growth and elongation (Aihemaiti et al., 2020), which restricted the water and minerals uptake, and initiated the physiological dysfunctions, resulting in growth and yield losses. Biochar and Si-NPs mitigated the V induced toxicity and enhanced the rice growth through distinct primary mechanisms. Biochar being a soil amendment immobilized the V through adsorption and complexation on its porous surface (**Figure 1**) and it also increased the soil pH (**Table 1**) which may have reduced the solubility of V. Thus, all these changes decreased V availability and protected rice plant from oxidative damages and resulted in improved minerals uptake and physiological functioning, leading to better growth and yield (Bashir et al., 2019; Tang et al., 2023). Silicon NPs primarily function as a physiological amendment. It is possible that rice plants took up the Si (**Supplementary Table S2**), which may have contributed to the deposition of silica in cell walls, creating a barrier that impeded the V translocation from roots to aerial parts. Moreover, Si-NPs also directly enhanced the chlorophyll contents, antioxidant activity, and osmolyte synthesis, which protected rice plants from oxidative damages, hence increasing growth and yield (Sharma et al., 2022). However, the differential response noted for different parameters can be linked to mechanistic divergences. We observed that the combination of BC and Si-NPs produced synergistic effects surpassing the individual effects. This synergy was linked with the ability of BC to immobilize V and reduce its availability. The reduced availability of V might then face the barrier induced by Si-NPs, which further reduced the uptake and accumulation of V in rice plants. Therefore, this two-layer defense improved the root growth, minerals uptake, plant functioning, and decreased the oxidative damages, contributing to better growth and yield.

**Figure 8:**
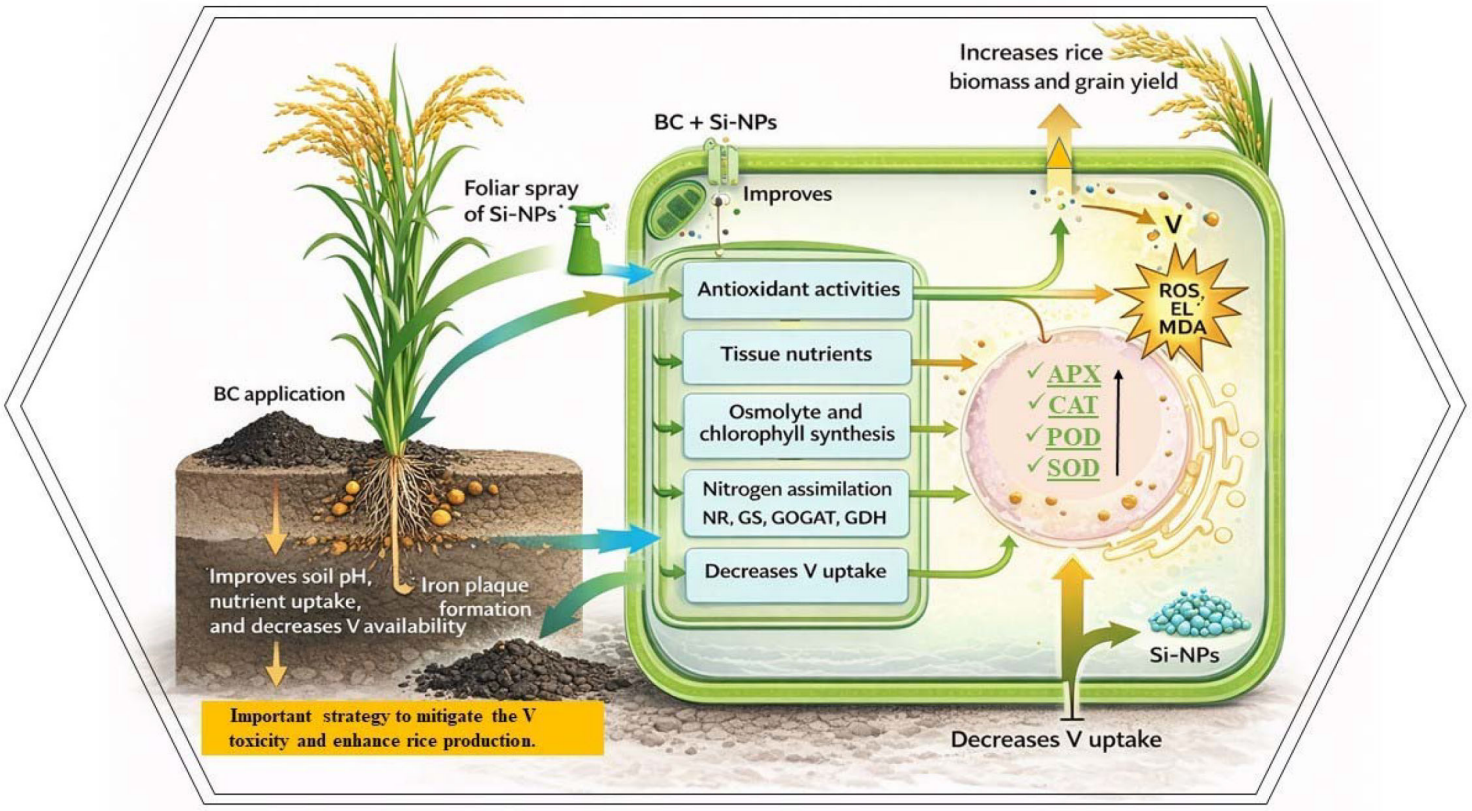
Schematic diagram for the role of BC and Si-NPs in mitigating the vanadium toxicity in rice.

Photosynthesis plays a crucial role in energy production and subsequent plant growth. In the present study, V toxicity severely decreased the photosynthesis as evidenced by a decrease in chlorophyll contents (Imtiaz et al., 2018). This aligns with previous studies reporting that V inhibits chlorophyll by increasing ROS production and damaging the enzymes and proteins involved in chlorophyll synthesis (Yuan et al., 2020; Zeeshan et al., 2020). However, BC and Si-NPs significantly enhanced chlorophyll contents. Biochar decreased the V availability and improved the nutrient homeostasis, and protected the cellular structure from oxidative damages contributing to better chlorophyll synthesis (**Figure 8**). Plants also uptake Si, which is deposited in leaf tissues and protects the chloroplast membranes from oxidative damage and results in better chlorophyll contents. Biochar and Si-NPs synergistically boosted the antioxidants activity, osmolytes synthesis, and decreases the V availability; thus, combined treatment showed more effective results in increasing chlorophyll contents.

Vanadium increased ROS, which damaged the cellular membranes and resulted in increased EL and MDA production (**Figure 2**). Rice plants enhanced antioxidant activity in response to V stress, aligning with previous studies showing that plants increase antioxidant activities as a defense to counteract oxidative damage (Mehmood et al., 2021). The activity of all the antioxidants was increased with BC and Si-NPs application. Different antioxidants, such as SOD, control the superoxide radicals, while POD safeguards the cellular membranes. Therefore, BC and Si-NPs mediated an increase in aforementioned activities, decreased the ROS production, aligning with previous outcomes (Altaf et al., 2025). The combination of BC and Si-NPs enhanced antioxidant activity, which decreased the H_2_O_2_, thus protecting the rice plant membranes as reported by a decrease in MDA and EL (**Figure 2**). These findings align with earlier outcomes showing that BC and NPs mitigate the membrane damage via increasing the antioxidant activity (Mehmood et al., 2020; Naeem et al., 2020). The reduction in EL in V stress plants with BC and Si-NPs is also linked with decreased ROS production and membrane preservation (Albqmi et al., 2023). Biochar and Si-NPs synergistically reduced the V availability and improved the nutrient and water status, and alleviated the increase in ROS production. This reduction in ROS production may have allowed rice plants to allocate resources more efficiently in maintaining the antioxidant system. It is also possible that BC acted as an upstream player to reduce the oxidative stress, while Si-NPs worked as downstream agents which enhanced the biochemical efficiency of plants in neutralizing the ROS. Thus, this double layer protection resulted in effective suppression of ROS, MDA, and EL.

Plants also accumulate different osmolytes, such as proline, soluble proteins, phenolics, and flavonoids, to counteract stress conditions (Hassan et al., 2025). In the current study, V stress decreased FA, SP, phenolics, and flavonoids, while enhancing proline, which favors the antioxidant activity, protects cellular membranes, and maintains osmotic adjustment (Raza et al., 2023). Furthermore, V toxicity decreased the SP and FA synthesis, likely due to disturbing the N metabolism and decreasing the N availability. Vanadium toxicity also decreased phenolic and flavonoid synthesis, while BC and Si-NPs enhanced their synthesis. The increase in phenolics and flavonoids eliminates the ROS production and alleviates the oxidative damage, thus ensuing better plant growth in stress conditions (Jan et al., 2023). Biochar and Si synergistically improved the soil N availability and retention, which ensured the better synthesis of FA and SP under V stress. It is also possible that BC and Si-NPs may have synergistically stimulated the phenylpropanoid pathway, thus contributing to an increase in phenolic and flavonoid production.

The results demonstrated that V accumulation followed a typical pattern, with maximum accumulation seen in roots rather than shoots. This inhibited translocation of V from roots to aerial parts is an important strategy to counteract V stress (Aihemaiti et al., 2020). The higher V concentration in roots may also linked with V interaction with polar substances present within the cell wall of roots (Altaf et al., 2020). Nutrients play an imperative part in plant functioning and membrane integrity (Liang et al., 2018; Nabaei and Amooaghaie, 2019). Vanadium toxicity reduced the nutrient accumulation in rice plants. This reduction might be linked with competition of V with nutrients for the same transport system (Imtiaz et al., 2016). This increased competition of V with nutrients decreases the nutrient uptake and their accumulation in stress conditions. Bichar and Si-NPs synergistically reduced the V accumulation in rice, which was linked with multi-stage synergistic mechanisms. The primary and dominant mechanism for reduction in V uptake was its immobilization in the soil rhizosphere. Biochar had higher surface area, porous structure, and functional groups (-OH, -COOH), which may have provided the active sites for adsorption and complexation of V, directly reducing its availability in soil pools (Jia et al., 2020). This processes was further enhanced by BC + Si-NPs, owing to their appreciable effects on soil pH, which might promote the V precipitation to less soluble forms, hence decreasing its uptake and accumulation by rice plants (Bashir et al., 2018). Silicon nano-particles also shield the plant roots and cover the root surface, which in turn decreases the metal uptake by roots, hence, contributing to a reduction in metals accumulation (Manzoor et al., 2022).

The second important mechanism contributed to the reduction in V translocation was the formation of iron plaque on root surfaces, which play a crucial role in mitigating the heavy metals toxicity (Khan et al., 2025). Iron plaque inhibits metal uptake and decreases their accumulation and subsequent toxic impacts on plants (Zheng et al., 2022; Khan et al., 2025). The combination of BC and Si-NPs significantly enhanced the Fe plaque formation on the root surface. Silicon improves the uptake and oxidation of iron in rice. Therefore, the Si-NPs may have promoted the release of oxygen and oxidants from the plant roots, which facilitated the precipitation of ferric iron as a plaque. This plaque works as a barrier and precipitates the metals at root surfaces, thus reduces its availability and uptake by plants (Zhang et al., 2021). The third possible mechanism was the growth dilution effect caused by BC and Si-NPs, where increased biomass led to lower accumulation of V in rice tissues. Biochar was rich in carbon (**Figure 1**), it increased the soil pH, nutrient retention, and immobilized the V, and created a less toxic environment for plant growth. Simultaneously, Si-NPs enhanced the iron plaque formation and improved the antioxidant activities, which allowed for improved growing conditions. Thus, this favorable growth environment led to an increase in biomass production and a decrease in V accumulation. Therefore, the decrease in V accumulation in rice by BC + Si-NPs is explained by synergistic sequences of V immobilization, iron plaque formation, and growth dilution impacts. However, future studies are needed on root transporter gene expression to underscore the exact mechanisms mediated by BC and Si-NPs for V competition and complexation. Rice plants uptake silicon as silicic acid via different transporters such as Lsi1 and Lsi2. These transporters are only linked with silicic acid, and they are not involved in the transport of V ions. Thus, direct competition for the same transporter, which was unlikely to be a mechanism for reduction in V uptake. The concentration of Si-NPs used here is within the range as suggested by the literature to induce positive impacts. Notably, the observed improvement in plant growth traits provides indirect evidence that the applied dose was not toxic. Furthermore, plants receiving the Si-NPs showed better growth under V, which also shows the benefits of stress alleviation surpassed any toxic impacts. The present study also does not assess the environmental risk assessment, including the long-term impacts of silicon nanoparticles on soil microbial communities and their bio-accumulation through the food chain. Thus, future studies should also prioritize the long-term field studies to investigate the impacts of silicon nanoparticles on soil microbes, food crops, and human health.

Nitrogen assimilation products are used in photosynthesis and play a crucial role in photosynthetic efficiency and plant growth. We found that V toxicity decreased the activity of NR, GS, GOGAT, and GH, which in turn decreased the N assimilation, its uptake, and accumulation in rice plants. This decrease in enzyme activity was linked with enhanced V accumulation and subsequent increase in ROS production. Conversely, BC and Si-NPs significantly enhanced NR, GS, GOGAT, and GH under V stress (**Figure 4**). The GS-GOGAT-GDH cycle is an important pathway involved in N assimilation. The cycle is initiated by the GS-catalyzed synthesis of glutamine and glutamate (Wang et al., 2020). The glutamine and glutamate produced after N assimilation function as intermediates such as tricarboxylic acid cycle components and chlorophyll precursors (Lea and Miflin, 2003). Biochar adsorbed the soil V, improved soil pH and nutrient availability, and decreased the oxidative stress on plants. This provided the favorable conditions to plants, resulting in improved activity of nitrogen assimilation enzymes. Moreover, silicon may have protected the cellular membranes from oxidative damages, this likely protected the nitrogen assimilation enzymes from oxidative damages. Thus, this protection improved the enzyme activity led to better N assimilation and rice growth under V stress. Biochar and Si-NPs also enhanced the accumulation of N in rice seedlings, which was likely due to up-regulation of the GS-GOGAT-GDH cycle (Yang et al., 2022). Biochar and Si-NPs also enhanced the accumulation of N in rice seedlings, which was likely due to up-regulation of the GS-GOGAT-GDH cycle (Yang et al., 2022).

## Conclusion

Vanadium stress impeded the rice biomass and yield by impairing the root growth, plant functioning, increasing the oxidative damages, and disturbing the nitrogen assimilation. Biochar and silicon nanoparticles application effectively mitigated the adverse impacts of vanadium and improved rice productivity. This was achieved through vanadium immobilization and reduction in its uptake through the formation of an iron plaque on the root surface. Biochar and silicon nanoparticles synergistically strengthened the antioxidant system, minimized the oxidative damages, consequently, improved the nitrogen assimilation and soil nutrient availability, supporting the plant performance. These results emphasized the suitability of biochar and silicon nanoparticles as an effective strategy to improve rice productivity in vanadium-polluted soils. However, our study has some limitations besides reporting the interesting findings. This study was performed in controlled pot conditions; therefore, field studies are needed under variable climate and soil conditions. Secondly, these findings were based on physiological and biochemical evidence; thus, transcriptome and metabolomics investigations are required to confirm the in-depth mechanism mediated by biochar and silicon nanoparticles to counteract vanadium toxicity. Long-term field trials are also necessary to evaluate the efficacy of biochar and silicon nanoparticles to determine their viability as a potential vanadium remediation strategy.

## Data Availability

The original contributions presented in the study are included in the article/**Supplementary Material**. Further inquiries can be directed to the corresponding author.
