# Supplementary material for: Biochar and nano-silicon partnership alleviates vanadium toxicity in rice through improving antioxidant defense, nitrogen assimilation and iron plaque formation
Source: Front Plant Sci. 2026 Mar 6;17:1778126. doi: 10.3389/fpls.2026.1778126 (PMC13002370; doi:10.3389/fpls.2026.1778126)
Supplement: Supplementary file 1 [file SupplementaryFile1.docx]

**Table S1:** Elemental composition of biochar and silicon nano-particles

| **Elemental composition of Biochar** | | |
| --- | --- | --- |
| **Element** | **Weight (%)** | **Atomic (%)** |
| C | 54.95 | 69.15% |
| N | 12.61 | 13.60 |
| Mg | 3.23 | 2.01 |
| Si | 25.98 | 13.98 |
| P | 0.26 | 0.13 |
| K | 0.93 | 0.36 |
| Ca | 2.03 | 0.77 |
| **Elemental composition of Silicon nano-particles** | | |
| C | 16.69 | 31.63 |
| N | 1.14 | 1.86 |
| Si | 81.26 | 65.85 |
| P | 0.87 | 0.64 |
| K | 0.03 | 0.02 |

**Table S2:** Effects of biochar and silicon nano-particles on silicon contents of rice plants grown under vanadium stress

| **Treatments** | **Root silicon (mg kg^-1^ DW)** | **Shoot silicon (mg kg^-1^ DW)** |
| --- | --- | --- |
| **Control** | 1.52±d | 0.57d |
| **V** | 1.06±d | 0.41d |
| **V + BC** | 8.19±b | 4.17b |
| **V + Si-NPs** | 7.20±c | 3.65c |
| **V + BC+ Si-NPs** | 9.24±a | 5.11a |

The presented data in the table is average of three replicates with ± SD and different letters showing the difference at p < 0.05 according to Tukey test (*p < 0.05*). V: vanadium, BC: biochar, Si-NPs: silicon nano-particles.
